# Lipids: A Suitable Therapeutic Target in Diabetic Neuropathy?

**DOI:** 10.1155/2017/6943851

**Published:** 2017-01-16

**Authors:** M. C. Perez-Matos, M. C. Morales-Alvarez, C. O. Mendivil

**Affiliations:** ^1^School of Medicine, Universidad de Los Andes, Bogotá, Colombia; ^2^Fundación Santa Fe de Bogotá, Department of Internal Medicine, Section of Endocrinology, Bogotá, Colombia

## Abstract

Diabetic polyneuropathy (DPN) encompasses multiple syndromes with a common pathogenesis. Glycemic control shows a limited correlation with DPN, arguing in favor of major involvement of other factors, one of which is alterations of lipid and lipoprotein metabolism. Consistent associations have been found between plasma triglycerides/remnant lipoproteins and the risk of DPN. Studies in cultured nerve tissue or in murine models of diabetes have unveiled mechanisms linking lipid metabolism to DPN. Deficient insulin action increases fatty acids flux to nerve cells, inducing mitochondrial dysfunction, anomalous protein kinase C signaling, and perturbations in the physicochemical properties of the plasma membrane. Oxidized low-density lipoproteins bind to cellular receptors and promote generation of reactive oxygen species, worsening mitochondrial function and altering the electrical properties of neurons. Supplementation with specific fatty acids has led to prevention or reversal of different modalities of DPN in animal models. Post hoc and secondary analyses of clinical trials have found benefits of cholesterol reducing (statins and ezetimibe), triglyceride-reducing (fibrates), or lipid antioxidant (thioctic acid) therapies over the progression and severity of DPN. However, these findings are mostly hypothesis-generating. Randomized trials are warranted in which the impact of intensive plasma lipids normalization on DPN outcomes is specifically evaluated.

## 1. Introduction

Diabetic neuropathy is a frequent and serious complication of both type 1 (DM1) and type 2 (DM2) diabetes. In patients with DM2, the prevalence of diabetic neuropathy has been estimated at 20–40% in different populations [1–3]. Diabetic neuropathy is a progressive, debilitating condition with a major impact on patient morbidity, mortality, and quality of life. There are five types of neurological syndromes related to diabetes mellitus: distal symmetric polyneuropathy (most frequent), autonomic neuropathy, small-fiber neuropathy (earliest), polyradiculopathy, and mononeuropathies [4, 5]. Despite important advances, results from observational studies and clinical trials suggest that other factors besides glycaemia play a large role in this particular complication.

## 2. Glycemic Control Is Not the Only Determinant of Diabetic Neuropathy

In the Diabetes Control and Complications Trials (DCCT), patients randomized to the intensive control arm achieved an HbA1c 1.8% lower than the conventional treatment arm after a follow-up period of 6.5 years and developed 69% less distal symmetrical polyneuropathy (DSP) (defined as DSP on physical examination plus abnormal nerve conduction in 2 different nerves or unequivocally abnormal autonomic test results) [6]. In the Epidemiology of Diabetes Intervention and Complications (EDIC) study, the original cohort of DCCT was followed observationally for another 8 years. The HbA1c difference between groups had entirely dissipated (8.0% prior intensive group versus 7.9% prior conventional therapy group) [7], yet the difference in diabetic polyneuropathy (DPN) incidence persisted (cumulative incidence 7% in the intensive group versus 3.5% control group). Furthermore, the NeuroEDIC study extended this follow-up for up to 14 years after the DCCT closure, and the between-group difference in the risk for neuropathy not only persisted but widened (25% in the former intensive group versus 35% in the former control group, *p* < 0.001) [8]. So the relevance of glycemic control in the progression of DPN in DM1 is paramount.

The Kumamoto and the Action to Control Cardiovascular Risk in Diabetes (ACCORD) trials found similar results in patients with DM2. In the Kumamoto study, patients treated with multiple insulin therapy (MIT) (3 or more daily administrations) achieved better glycemic control than those under conventional insulin therapy (HbA1c 7.1% MIT group versus 9.4% conventional therapy, *p* < 0.05). This better glycemic control translated into less nerve damage after 6 years, with a small but significant difference (median nerve conduction velocity [NCV] 53.2 m/s in MIT versus 50.2 m/s in conventional group, *p* < 0.05) [9]. Similarly, in the glycemic component of the ACCORD trial, patients originally randomized to strict glycemic control (HbA1c at glycemic component discontinuation 6.4%) had a slower progression of DPN versus the standard treatment group (HbA1c 7.5%) (hazard ratio [HR] for loss of ankle jerk at study end 0.90, 95% CI: 0.84–0.97, *p* = 0.005) [10].

Nonetheless, not all outcome studies in DM2 have found a significant impact of glycemic control on neuropathy. A very large difference in final HbA1c (8.4% in control group versus 6.9% in intensive group) had no impact on the cumulative incidence of any type of neuropathy in the Veterans Affairs Diabetes Trial (VADT) (43.5% control group, 43.8% intensive group) [11]. The United Kingdom Prospective Diabetes Study (UKPDS) of intensive treatment with sulphonylureas or insulin versus standard therapy in patients with DM2 produced comparable findings. Despite better HbA1c control (7.0% in intensive arm versus 7.9% in standard arm, *p* < 0.001), incidence of DSP measured by absent ankle reflexes did not differ between groups (35% in the intensive treatment group versus 37% in the standard treatment group, *p* = 0.60) [12].

Finally, the Action in Diabetes and Cardiovascular Disease: Preterax and Diamicron Modified Release Controlled Evaluation (ADVANCE) study showed a benefic effect of intensive glycemic control on nephropathy (HR 0.79, IC 95%: 0.66–0.93) but no significant effect on either retinopathy or neuropathy in patients with DM2 [13]. Two recent large cardiovascular outcome trials in patients with DM2 (LEADER with the Glucagon Like Peptide-1 [GLP-1] agonist liraglutide and EMPA-REG with the Sodium-Glucose Cotransporter-2 [SGLT-2] inhibitor empagliflozin) found significant reductions of cardiovascular risk with newer oral antidiabetic therapies but did not report on neuropathy endpoints [14, 15].

So hyperglycemia does not seem to be the sole factor explaining the appearance and progression of DPN, and the effect of glycemic control on the improvement of DPN appears to be variable, particularly among patients with DM2.

Diverse pathophysiological mechanisms have been implicated in the development and progression of DPN. The high oxidative stress characteristic of hyperglycemia exerts injury to nerve cells through lipid peroxidation, direct damage to DNA with pathological activation of repair pathways, depletion of cellular antioxidants, and induction of proinflammatory transcription factors [16]. Another pathway leading from hyperglycemia to DPN entails the activation of the intracellular enzyme aldose reductase, which transforms glucose that has not been oxidized via glycolysis into sorbitol and fructose. This so-called polyol pathway leads to the intracellular accumulation of osmotically active sorbitol, causing cellular edema and loss of important metabolic mediators like taurine, myoinositol, and adenosine. Also, the reaction catalyzed by aldose reductase utilizes NADPH, so this pathway depletes the cell of NADPH, necessary for the regeneration of glutathione, the main defense against oxidative damage [17]. Local alterations of nociceptors and neural growth factors (neurotrophins) also play a role, especially in painful DPN. Chronic and continuous stimulation of the nociceptor transient receptor potential cation channel subfamily V member 1 (TRPV-1) in early DPN leads to local release of various growth factors, importantly NGF (nerve growth factor) and brain-derived neurotrophic factor (BDNF). This creates a feedforward loop in which NGF binds to the trkA receptor, which lowers the threshold for TRPV-1, leading to further sensitization and pain and to further NGF release [18]. The activation of certain isoforms of protein kinase C is characteristic of diabetic complications, and it is presumed to be involved in DPN as well [19]. Protein kinase C is a second messenger kinase that activates nuclear factor kappa-B (NF kappa-B) and other proinflammatory transcription factors.

The hexosamine pathway may also contribute to diabetic neuropathy. When cells have a high glucose influx, some of the fructose-6 phosphate in the glycolytic pathway is diverted by glutamine:fructose-6-phosphate transferase to glucosamine-6 phosphate. This hexosamine is used to produce UDP-N-acetyl glucosamine (UDP-GlcNAc). UDP-GlcNAc is then enzymatically added to the serine and threonine residues of multiple transcription factors, modifying their activity. Involvement of this pathway has been clearly demonstrated for other diabetic complications [20], but its role in DPN is less clear. Yet another plausible mechanism of nerve dysfunction in diabetes involves the nonenzymatic glycation of cellular proteins. Chronic elevation of glucose in the cellular milieu facilitates the formation of advanced glycation end-products (AGEs), which directly hinder the function of multiple essential cellular and extracellular proteins (tubulin, actin, and laminin). AGEs also bind to and activate a specific receptor (the receptor for AGEs or RAGE), inducing a proinflammatory, prooxidative transcriptional program in peripheral nerves [21].

However, despite these well-known pathogenic mechanisms of glucose burden on DPN, the same has not been consistently replicated in various clinical trials as mentioned. This suggests the role of additional factors which might influence the appearance and progression of DPN. Here, we propose that alterations of lipid metabolism (which are very frequent in patients with DM2 and/or the metabolic syndrome) participate in several key pathways of DPN pathogenesis and that normalization of lipid metabolism may constitute an appealing target for the prevention or treatment of DPN.

## 3. Plasma Lipids Are Associated with Progressive DPN

In several large observational studies, an interesting observation has been the baseline between-group differences in lipid profile in patients with DM2 who go on to develop DPN and those who do not. In the European Diabetes Prospective Complications (EURODIAB) study of patients with DM1, total cholesterol (TC), LDL cholesterol (LDLc), and TG levels were significantly associated with incident DPN over a 7.3-year follow-up (Odds Ratio [OR] 1.26, *p* = 0.001; OR: 1.22, *p* = 0.02; and OR 1.35, *p* < 0.001, resp.), even after adjustment for baseline HbA1c and diabetes duration [22]. Concordantly, in a 52-week prospective study of patients with DM2, plasma triglycerides (TG) were associated with progressive DPN, defined as a loss of more than 500 fibers/mm^2^ in a sural nerve biopsy (*p* = 0.04 for plasma TG difference between progressors and nonprogressors) [23]. Likewise, the Utah Diabetic Neuropathy study found an association between plasma triglycerides ≥150 mg/dl and the risk of DPN at entry in patients with DM2 (relative risk [RR]: 2.3, 95% CI: 1.1–4.7) [24]. Also, LDL particle size as a marker of atherogenic dyslipidemia appears to be an independent risk factor for neuropathy [25], and patients with mixed dyslipidemia have been shown to exhibit prolonged cutaneous silent period latency, a measure of small-fiber neuropathy [26].

## 4. Mechanisms Linking Lipids to Diabetic Neuropathy

### 4.1. Peripheral Nerves Are Affected by Insulin Resistance

Even though glucose uptake in the nervous system is largely insulin-independent, there is evidence of insulin signaling in peripheral nerves [27]. Insulin signaling in peripheral neurons proceeds in a way analog to that in other cells, with successive phosphorylation of the insulin receptor itself, then the insulin receptor substrate 2 (IRS-2), phosphoinositide 3-kinase (PI3K), phosphoinositide-dependent kinase-1 (PDK1), and subsequently protein kinase B (PKB/Akt) [28]. Direct insulin administration of insulin at doses insufficient to change plasma glucose was able to prevent and reverse features of diabetic neuropathy (motor conduction velocities and axonal atrophy) in the sural nerves of streptozotocin-induced diabetic mice [29]. Studies in obese diabetic ob/ob mice have demonstrated a lack of PKB/Akt activation in peripheral nerves in response to direct (intrathecal) administration of insulin [30]. Hyperglycemia may directly affect the neural response to insulin. In vitro studies of the impact of insulin on the nerve action potential under normal or high glucose conditions have found that hyperglycemia prolongs the action potential, an effect that is abolished by insulin [31]. However, under normoglycemic conditions the effect of insulin was to reduce the conduction velocity of oxygenated nerves. Furthermore, in vitro studies have shown that continuous exposure to high insulin concentrations abolishes the ability of acute insulin exposure to activate the Akt signaling pathway in dorsal root ganglion neurons [32]. Thus, the hallmarks of molecular resistance to insulin action in other tissues (adipose and liver) are also present in nerve tissue. Human patients with the metabolic syndrome are characterized by insulin resistance and a chronic low-degree inflammation status [33, 34]. In these patients, insulin resistance assessed through the homeostatic model assessment–insulin resistance (HOMA-IR) index has shown a positive and independent association with clinical scores of peripheral neuropathy (Odds Ratio: 1.2 per unit, 95% CI: 1.1–1.4) [35].

### 4.2. Free Fatty Acids Mediate Insulin Resistance and Dysfunction in Peripheral Nerves

High plasma levels of free fatty acids (FFA) are a hallmark of insulin resistance. Decreased inhibition of adipocyte hormone-sensitive lipase due to insulin resistance leads to a continuous release of FFA [36]. FFA in turn perpetuate and worsen insulin resistance in adipose and other tissues by inducing intracellular formation of diacylglycerol and ceramides that activate protein kinase C-theta and delta isoforms (PKC-theta and PKC-delta) and serine-threonine kinases that phosphorylate IRS and reduce their signaling capacity [37]. On the other hand, the phospholipid bilayer of cells from healthy patients is characterized by a high concentration of polyunsaturated fatty acids (PUFA), a composition that facilitates insertion of membrane receptors and transporters and uptake of external substrates. In DM2, increased FFA lead to high cytoplasmic saturated fatty acyl-CoA, which allosterically inhibits fatty acid desaturases and reduces synthesis of PUFA [38]. Under these circumstances, membrane flexibility decreases and multiple functions associated with electrical conduction and signal transduction may become affected [39]. A rigid membrane increases oxidative stress and further induces insulin resistance by its limited capability glucose transporter (GLUT) expression. High intracellular saturated FFA levels also activate nuclear factor kappa-B (NF-kB) signaling by directly stimulating expression of the p65 subunit of NF-kB [40]. This pathway raises production of reactive oxygen species (ROS) and promotes oxidative stress, which is a central factor in the appearance and progression of DPN [41].

In streptozotocin diabetic rats, 5 weeks of supplementation with PUFAs gamma-linolenic (omega 6) and eicosapentaenoic (omega 3) acids led to a significant decrease in the progression of DPN measured as sensitive and motor NCV [42]. A multicenter clinical trial revealed a significant improvement of 13 DPN parameters (including conduction velocities, thermal sensitivity, and tendon reflexes) in DM2 patients supplemented with gamma-linolenic acid for 1 year [43]. A recent study focused on the causality of the association between FFA and DPN. Patients with DM2 received an intralipid and heparin infusion to intentionally raise FFA levels and had their heart rate variability measured by spectral analysis for 3 hours. Plasma FFA correlated positively with the low frequency/high frequency variability ratio (higher values indicate lower heart rate variability) (*r* = 0.57, *p* < 0.02). After three months of good glycemic control, when circulating FFA had dropped to normal levels, heart rate variability measures also returned to normal [44].

### 4.3. Imbalance of Mitochondrial Bioenergetics Further Mediates Neuropathy

Cellular energy metabolism is centered at the mitochondria, which is consequently the main site of reactive oxygen species (ROS) generation. In neurons and glial cells, a dysregulation of mitochondrial bioenergetics as seen in DM2 has been associated with abnormal increases in mitochondrial fission and biogenesis [45]. Mitochondria shift their balance from fatty acid biosynthesis towards continued oxidation, using for this purpose most available acyl-carnitines and depleting a key substrate for myelin lipid biosynthesis [46]. Derangement of substrate utilization may lead to increased production of mitochondrial ROS, release of cytochrome C, and activation of proapoptotic pathways leading to neuronal damage [46, 47].

Transcriptional, proteomic, and functional changes indicative of altered mitochondrial substrate utilization associated with greater ROS generation and less respiratory capacity in the context of insulin-resistant diabetes have been detected in heart [48], skeletal muscle [49, 50], and sensory neurons [51].

FFA have the ability to directly inhibit the respiratory chain [52–54], a property that has been demonstrated in Schwann cells in vitro [55]. A study in streptozotocin diabetic rats found that insulin doses insufficient to induce changes in plasma glucose were still able to normalize rates of mitochondrial coupled respiration in cells from dorsal root ganglia [56]. Murine models of DM2 display reduced glycolytic intermediaries in peripheral nerves and dorsal root ganglia, in association with increased oxidative damage of proteins and lipids [57]. These changes appear to affect first neurons from longer peripheral nerves, like the sciatic nerve [58]. Mechanistically, AMP-activated protein kinase (AMPK) and peroxisome proliferator-activated receptor gamma coactivator 1-alpha (PGC-1 alpha) are “central hubs” of energy metabolism [59, 60] that appear to be involved in the pathway from fatty acids to mitochondrial dysfunction and DPN. A high-fat diet increases mitochondrial concentrations of fatty acid oxidation intermediaries and decreases PGC-1 alpha expression in skeletal muscle [61]. A cross-sectional study comparing gene expression patterns in skeletal muscle biopsies from patients with insulin resistance, patients with DM2, and controls found a significant downregulation of PGC-1-responsive genes involved in mitochondrial ATP production in the first two groups [62]. The expression of PGC-1 and nuclear respiratory factor-1 (NRF-1) responsive oxidative metabolism genes is reduced in muscle tissue of patients with DM2 and in normoglycemic relatives of patients with diabetes [50]. Interestingly, stimulation of AMPK signaling has improved neuropathic manifestations like thermal hypoalgesia in a rodent model of diabetic neuropathy [63]. Administration of troglitazone (a PPAR-gamma agonist) to diabetic obese rats improved NCV [64].

### 4.4. Oxidized Lipids May Promote DPN

Increased LDL cholesterol and TG levels have been shown to be associated with a faster progression to end-stage renal disease, blindness and peripheral neuropathy in patients with DM2 [65]. The mechanism behind the LDL-DPN relationship is thought to reside in increased oxidative environment, as explained above. In fact, oxidation of LDL cholesterol is increased in patients with diabetes compared to healthy controls [66], resulting in a proinflammatory state. Dorsal root ganglia express the lectin-like oxLDL receptor (LOX-1). When oxidized LDL (oxLDL) bind to this receptor, a signaling pathway is activated that increases ROS and oxidative stress. The same process occurs in the nerve roots of patients with DPN, particularly via activation of NADPH oxidase, before a significant impairment of glycemia becomes evident [67].

### 4.5. Atypical Sphingolipids, Another Metabolic and Neurotoxic Link?

Sphingolipids are a class of naturally occurring lipids made by subsequent modifications of a sphingoid base, mostly sphingosine [68]. The rate-limiting step in their synthesis is the condensation of L-serine and palmitoyl-CoA, catalyzed by the enzyme serine-palmitoyl transferase (SPT) [69]. Complex lipids from this group such as ceramide and sphingomyelin are involved in cell structure and signaling [68]. Deoxy-sphingolipids (DOSL) are atypical sphingolipids characterized by the lack of an OH group in C1. Several DOSL display neurotoxic activity [70]. DOSL are produced when SPT activity is altered and it uses L-alanine or glycine instead of serine as amino acid substrate [68]. As serine and alanine are involved in carbohydrate metabolism, it is believed that DOSL synthesis is a metabolic intersection between lipid, carbohydrate pathways, and oxidative stress [71], especially in patients with DM2 [72].

Observational studies have demonstrated that DOSL levels are increased in patients with metabolic syndrome and/or DM2. A study comparing the sphingolipid profile of patients with DM1, DM2, and controls found increased levels of DOSL in patients with DM2 (0.05, 0.09, and 0.05 arbitrary units, resp.) [71]. In a case-control study, patients with DM2 also had higher DSOL plasma levels compared to controls (0.19 microM and 0.12 microM, resp., *p* = 0.005) [72]. Plasma sphingolipid profiling of patients with DPN compared to other types of neuropathy and patients without neuropathy reveals increased atypical sphingolipids (0.11 microM DPN versus 0.06 microM controls, *p* < 0.001) [73]. In a subgroup study from EDIC, patients who reported neuropathy at any point of follow-up exhibited higher deoxy-ceramide levels than those without neuropathy (12.3 versus 10.6, *p* = 0.049 units/curve area) [74]. A pilot model with diabetic rats demonstrated that intentionally lowering plasma DOSL may improve neuropathy measures like mechanical sensitivity and NCV [75]. In a trial comparing treatment with fenofibrate versus niacin for 6 weeks in patients with primary hypercholesterolemia or mixed dyslipidemia, fenofibrate effectively lowered atypical sphingolipids (0.13 microM before, 0.09 microM after treatment, *p* ≤ 0.001) [76], which suggests that PPAR-alpha agonists may provide a positive impact on DPN (see below). The mechanism of DOSL-induced neurotoxicity remains to be elucidated.

**Figure 1:**
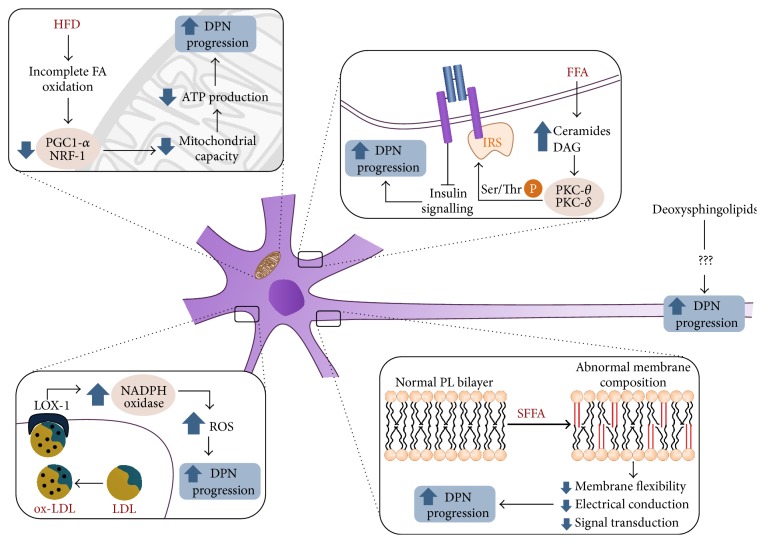
Pathogenic mechanisms linking abnormal lipid metabolism to progression of diabetic neuropathy. HFD: high-fat diet, FA: fatty acids, PGC-1alpha: PPAR-gamma coactivator 1-alpha, NRF-1: nuclear respiratory factor-1, DPN: diabetic polyneuropathy, FFA: free fatty acids, IRS: insulin receptor substrates, PKC-theta: protein kinase C, theta isoform, PKC-delta: protein kinase C, delta isoform, Ser/ThrP: phosphorylation in serine or threonine, ox-LDL: oxidized LDL, LOX-1: lectin-like oxidized LDL receptor, NADPH oxidase: reduced nicotinamide-adenine dinucleotide phosphate oxidase, PL: phospholipid, and SFFA: saturated free fatty acids. Insulin resistance or a high-fat diet increase the cellular supply of FFA, leading to decreased expression of PGC-1alpha and NRF1-alpha-responsive genes and subsequently to impaired mitochondrial capacity and nerve dysfunction. Increased supply of FFA also causes uncontrolled formation of DAG and ceramides, which activate atypical PKC isoforms and promote serine/threonine phosphorylation of IRS, decreased insulin signaling, and defective nerve growth and repair. The augmented availability of SFFA in insulin resistance leads to changes in the fatty acid composition of plasma membrane phospholipids. Membranes richer in saturated FA are more rigid and exhibit disturbances of electrical conduction and a reduced capacity for receptor expression and signal transduction, all of which worsen DPN. Accelerated ROS production in diabetes generates oxLDL that bind to the LOX-1 receptor and activate NADPH oxidase, worsening ROS production even further and hastening the progression of DPN. Finally, oxidized deoxysphingolipids are neurotoxic lipids associated with DPN, but their mechanism of action is still unknown.

The mechanisms linking deranged lipid metabolism to DPN are summarized in Figure 1.

## 5. Treatment of Dyslipidemia: Its Impact on DPN

### 5.1. Triglyceride-Reducing Therapy

Fibrates are a class of lipid-lowering therapies with demonstrated efficacy at reducing TG and increasing HDLc in patients with DM2. Recent evidence suggests a positive effect of fibrates on DPN progression. In a report from the Fremantle Diabetes Study that included 531 patients with DM2 followed for 5 years using either statins or fibrates as lipid-lowering therapy, treatment with fenofibrate was associated with a significant decrease in the appearance of neuropathy (measured by the Michigan Neuropathy Scoring Instrument (MNSI) [HR 0.52, *p* = 0.042]) [77]. In the Fenofibrate and Event-Lowering in Diabetes (FIELD) study of 9795 patients with DM2 who were randomized to fenofibrate or placebo, the fenofibrate group had a significantly lower rate of nontraumatic amputations (HR 0.62, *p* = 0.011) [78]. Mechanistic studies in obese db/db mice have found that fenofibrate markedly activates the above-mentioned PPARalpha-AMPK-PGC1 pathway in the sciatic nerve, while improving the animals' tactile threshold [79].

Omega-3 fatty acids are essential polyunsaturated fatty acids, a group that includes eicosapentaenoic and docosahexaenoic acids (DHA). In patients with DM2, plasma levels of omega-3 acids correlate negatively with insulin resistance and dyslipidemia [80]. Experimentally, an increased production of omega-3 in a diabetic mice model confers resistance to diet induced obesity and diabetes [81]. Supplementation with fish oil containing DHA completely prevented the development of neuropathy in streptozotocin-induced diabetic mice [82] and led to preservation of NCV and Na+/K+ ATPase activity in sural nerve of streptozotocin-induced diabetic rats [83]. The polyunsaturated and anti-inflammatory nature of omega-3 may be key to these effects against diabetes-induced nerve dysfunction.

### 5.2. Cholesterol-Lowering Therapy

Statins are the cornerstone of hypercholesterolemia management. By inhibiting the rate-limiting enzyme in the cholesterol biosynthesis pathway (conversion of hydroxymethylglutaryl CoA to mevalonate), they also prevent the formation of isoprenoid intermediaries like isopentenyl-pyrophosphate, dimethylallyl-pyrophosphate, geranyl-pyrophosphate, and farnesyl-pyrophosphate. Isoprenoids play an important role in the posttranslational modification and membrane attachment of multiple signaling molecules, among them GTP-binding proteins of the Ras and Rho family. Therefore changes in the availability of farnesyl-PP (associated with Ras proteins) or geranyl-PP (associated with Rho proteins) affect a great number of cellular processes beyond cholesterol production [84]. Streptozotocin-induced diabetic mice showed normalization of their NCV of the saphenous and sciatic nerves after 2 weeks of treatment with 0.3–20 mg/kg of rosuvastatin and a normalization of thermal hyperalgesia with the 20 mg/kg dose. These results indicated improvement in both large and small nerve fibers. The complete reversal of these effects with mevalonate supplementation implies that they were mediated by reduced production of isoprenoid precursors [85].

There is also evidence of DPN improvement with cholesterol-lowering therapies such as statins or ezetimibe in clinical studies. In the Fremantle Diabetes Study, patients with DM2 treated with statins evidenced a 35% reduction in the incidence of DPN [77]. A recent study demonstrated that patients with DM2 treated with simvastatin + ezetimibe or rosuvastatin had lower lipid peroxidation (LPO) markers versus placebo and a significant reduction in the Neuropathy Symptoms Score from baseline [86], lending further support for this pathway as a pharmacological target in DPN.

### 5.3. Lipoic Acid as a DPN Therapy

In the context previously described, current approaches for DPN therapy include molecules with antioxidant properties [87]. Lipoic acid or thioctic acid (LA) is an octanoic acid derivative that has been used for symptomatic relief in diabetic polyneuropathy with positive results. Three pathways may explain its effect: (1) LA has the capacity to directly scavenge reactive oxygen species; (2) LA regenerates endogenous antioxidants (glutathione, vitamin E, vitamin C, and coenzyme Q); and (3) LA has metal chelating activity over iron and copper. Several clinical trials have provided evidence of the efficacy of LA against neuropathy in patients with DM1 and DM2. A recent meta-analysis of 15 randomized controlled trials evaluating the efficacy of LA administration on improvement of objective DSP measures found a positive effect on peripheral NCVs with the 300–600 mg i.v. dose for at least 3 weeks (OR 4.03, 95% IC 2.73–5.94), with no significant adverse effects [88].

## 6. Summary/Conclusion

DPN is a frequent, serious, and debilitating chronic complication of diabetes mellitus. Despite its relevance, very little is known about the details of its molecular pathogenesis and consequently the availability of targeted, efficacious therapies is limited. Alterations in the metabolism of lipids including triglycerides, cholesterol, fatty acids, and sphingolipids have been implicated in the pathogenesis of DPN and constitute an interesting molecular target for the treatment of clinical DPN. However, most of the available evidence in this respect is mechanistic (i.e., animal on in vitro studies) or from observational human studies.

The evidence from secondary or post hoc analysis of randomized trials is limited by patient heterogeneity, variations in dose and follow-up duration, and particularly the methodology used to define DPN. Most studies have used sign-driven scales (like the Michigan Neuropathy Score), symptom-driven scales (like the Total Symptom Score), or vibration perception thresholds in an attempt to make DPN a measurable, comparable variable, but only a few have measured NCV, a truly objective measure of nerve functionality. Furthermore, it is known that small-fiber neuropathy, the earliest manifestation of DPN, can be missed by all these methodologies. For that reason a group of new techniques for DPN diagnosis have come into place, including corneal confocal microscopy, laser Doppler image flare, sudomotor reflex assessment, quantitative sensory testing, and skin biopsy [89]. These small-fiber neuropathy detection tools should be incorporated into endpoint ascertainment in future studies of lipids and DPN.

In summary, DPN is a complex and multifactorial entity in which various factors besides hyperglycemia play an important role. There is a host of indirect evidence showing that deranged lipid metabolism at the cellular and whole-organism level aggravates or perpetuates DPN, and mitigation of such alterations improves DPN in animal models of diabetes.

In consonance with these observations, clinical trials in which lipid-modifying therapies have been assessed for their impact on cardiovascular morbidity and mortality have shown as descriptive findings positive effects on DPN, but the available evidence is insufficient to solidly implicate lipids as a pharmacological target in DPN.

Future research should concentrate on targeting lipids with one or more aggressive interventions specifically in patients with DM2 whose DPN is detectable but whose progression can still be largely prevented. Such studies could have selection criteria focused on the presence and severity of DPN instead of plasma lipid concentrations. Until then, careful control and follow-up of plasma lipids in patients with diabetes can only be considered an adjunct strategy against DPN.
